# Self-Healing Performance of Smart Polymeric Coatings Modified with Tung Oil and Linalyl Acetate

**DOI:** 10.3390/polym13101609

**Published:** 2021-05-17

**Authors:** Norhan Ashraf Ismail, Adnan Khan, Eman Fayyad, Ramazan Kahraman, Aboubakr M. Abdullah, Rana Abdul Shakoor

**Affiliations:** 1 Center for Advanced Materials, Qatar University, Doha 2713, Qatar; ni1300925@qu.edu.qa (N.A.I.); ak1704740@qu.edu.qa (A.K.); emfayad@qu.edu.qa (E.F.); bakr@qu.edu.qa (A.M.A.); 2 Department of Chemical Engineering, College of Engineering, Qatar University, Doha 2713, Qatar

**Keywords:** microcapsules, epoxy, coatings, self-healing, electrochemical impedance

## Abstract

This work focuses on the synthesis and characterization of polymeric smart self-healing coatings. A comparison of structural, thermal, and self-healing properties of two different polymeric coatings comprising distinct self-healing agents (tung oil and linalyl acetate) is studied to elucidate the role of self-healing agents in corrosion protection. Towards this direction, urea-formaldehyde microcapsules (UFMCs) loaded with tung oil (TMMCs) and linalyl acetate (LMMCs) were synthesized using the in-situ polymerization method. The synthesis of both LMMCs and TMMCs under identical experimental conditions (900 rpm, 55 °C) has resulted in a similar average particle size range (63–125 µm). The polymeric smart self-healing coatings were developed by reinforcing a polymeric matrix separately with a fixed amount of LMMCs (3 wt.% and 5 wt.%), and TMMCs (3 wt.% and 5 wt.%) referred to as LMCOATs and TMCOATs, respectively. The development of smart coatings (LMCOATs and TMCOATs) contributes to achieving decent thermal stability up to 450 °C. Electrochemical impedance spectroscopy (EIS) analysis indicates that the corrosion resistance of smart coatings increases with increasing concentration of the microcapsules (TMMCs, LMMCs) in the epoxy matrix reaching ~1 GΩ. As a comparison, LMCOATs containing 5 wt.% LMMCs demonstrate the best stability in the barrier properties than other developed coatings and can be considered for many potential applications.

## 1. Introduction

The properties and performance of materials are strongly affected by environmental changes. A dangerous environmental phenomenon that harms materials is corrosion. Corrosion is one of the world’s most significant problems, especially in the industrial field, which results in equipment damage and production discontinuity. Furthermore, corrosion is responsible for about 70% of the repair/replacement needs in pipelines and piping (process equipment on platforms). Operation and maintenance costs represent around 52% of the total expenses; out of these, 80% are due to corrosion [1]. Organic coatings are the oldest, most common, and efficient option for metal protection and corrosion delaying. Organic coatings can be polymer mixtures, fluid carriers, colors, corrosion inhibitions, and additives [2]. However, this type of coatings’ main disadvantage is the high chance of corrosion owing to the initiation of mechanical stimulus or scratch, facilitating the penetration of corrosive media, such as acid rain, seawater, and industrial wastes, into the metal surface [3]. The above-stated problem is inherently due to the low hardness of the polymeric matrix. The polymeric coatings’ limitations can be addressed by considering a new generation of coatings, referred to as smart self-healing coatings. By definition, self-healing is the material’s ability to automatically heal its damages without any external support or aid. Smart coatings contain unique properties that allow them to feel the surrounded environment and adapt themselves to give a proper response to any stimulus [4]. There are many types of smart coating systems—antimicrobial, conductive, antifouling, self-healing, self-cleaning, etc. [4,5,6,7]. The material selection challenge is to choose a material capable of demonstrating high safety, long lifetime, and low maintenance costs [8]. Since the investigation of a perfect material is not a realistic idea, the self-healing technique can be considered as a practical alternative. Compared with conventional coatings, smart self-healing coatings can restore their properties to resist corrosion damage due to unexpected stimulus [3,9].

Among numerous types of smart coatings, coatings containing microcapsules are promising. The self-healing mechanism in the smart coatings containing microcapsules is based on the release of the self-healing agent loaded into the microcapsules due to the initiation of any sudden stimulus on the surface of the coated substrate [10,11]. The microcapsules can have different formulations such as urea-formaldehyde microcapsules (UFMCS) [12,13,14] or multilayered urea-formaldehyde microcapsules (MLUFMCs) [15], melamine urea-formaldehyde microcapsules (MUFMCs) [12], or polyurethane microcapsules (PUMCs) [16]. Furthermore, the microcapsule shell can contain different types of self-healing agents such as tung oil [17], linseed oil [18], silane [19], linalyl acetate [20], or dicyclopentadiene (DCPD) [21] that has been successfully reinforced into the epoxy matrix [22]. Different studies conclude that epoxy resins are preferred over other polymers for coating applications due to their salient characteristics such as lightweight, high thermo-mechanical performance, excellent adhesion, good chemical resistance, and corrosion resistance [22]. There is no study on the concentration effect on self-healing performance of microcapsules in the coating matrix; in addition, no research was found to explain the comparative self-healing capabilities of these healing species.

In this work, urea-formaldehyde microcapsules (UFMCS) were synthesized using an in-situ emulsion polymerization method [23]. The prepared urea-formaldehyde microcapsules were separately loaded with two different self-healing agents (tung oil and linalyl acetate) referred to as tung oil-modified microcapsules (TMMCs), and linalyl acetate modified microcapsules (LMMCs), respectively. Urea-formaldehyde microcapsules were selected as containers or shells due to their availability, low cost, high heat capacity, and good thermal stability [12]. Tung oil and linalyl acetate were chosen as self-healing agents because of their promising self-healing ability [24,25,26]. The TMMCs (3 wt.% and 5 wt.%), and LMMCs (3 wt.% and 5 wt.%) were separately reinforced into the epoxy matrix to develop TMMCs and LMMCs reinforced smart coatings (TMCOATs, and LMCOATs) respectively. For an accurate comparison, epoxy coatings without any reinforcement referred as reference coatings were also developed. A comparative study concludes that both types of smart coatings (TMCOATs and LMCOATs) exhibit improved self-healing performance compared to the reference coatings.

## 2. Experimental Section

### 2.1. Materials

The chemicals used in the synthesis of loaded urea-formaldehyde microcapsules includes deionized water, ethylene maleic anhydride copolymer (EMA), urea, ammonium chloride, resorcinol, 37 wt.% formaldehyde, tung oil and linalyl acetate (as self-healing agents), hydrochloric acid, and sodium hydroxide (for adjusting the pH of the urea solution), were purchased from Sigma Aldrich, Darmstadt, Germany. The materials used for preparing the coating samples including epoxy resin (Epon resin 815 C), diethylenetriamine (as an epoxy hardener), ethanol (for cleaning the carbon steel surface before coating) were also purchased from Sigma Aldrich, Darmstadt, Germany while the carbon steel sheets as substrates were purchased from local source. The 0.1 M NaCl solution was placed inside the Gamry cell (as a corrosive media) to which the synthesized coatings were exposed to evaluate their corrosion protection performance. All the chemicals were purchased from Sigma Aldrich, Darmstadt, Germany, except the steel substrates. 

### 2.2. Synthesis of Urea-Formaldehyde Loaded Microcapsules

**Figure 1:**
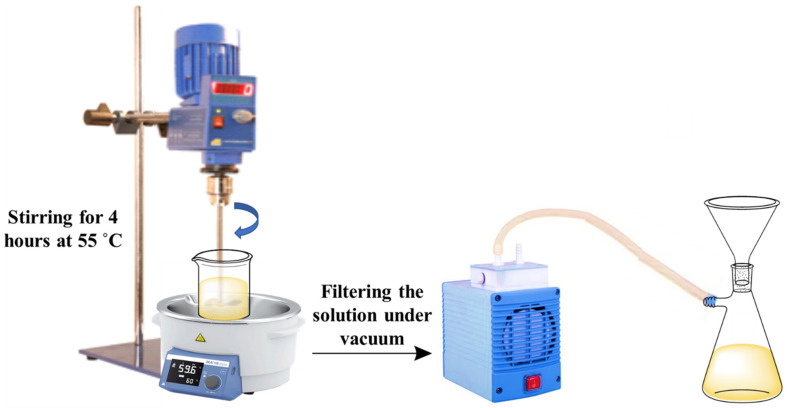
Schematic diagram illustrating the setup for the synthesis of urea-formaldehyde microcapsules (UFMCs) loaded with tung oil and linalyl acetate.

Urea-formaldehyde microcapsules (UFMCs) loaded with tung oil (TMMCs) and linalyl acetate (LMMCs) were synthesized using an in-situ emulsion polymerization method [23]. Figure 1 schematically represents the experimental setup used to develop TMMCs and LMMCs.

**Figure 2:**
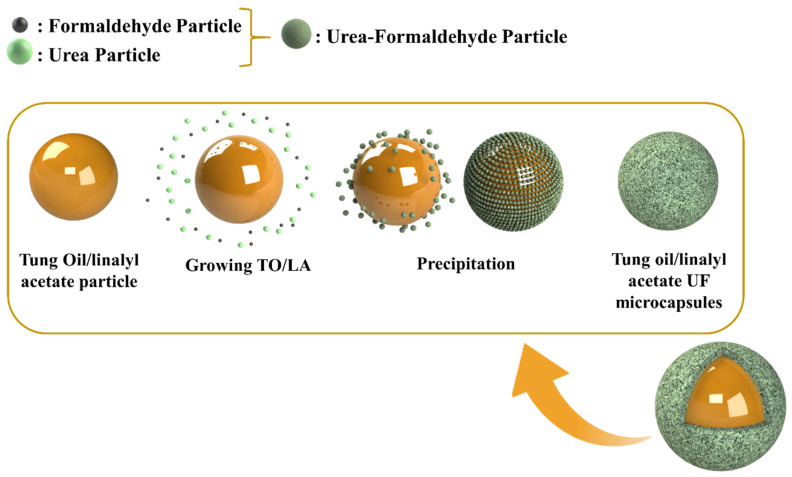
Schematic illustration of steps for the synthesis of urea-formaldehyde microcapsules loaded with tung oil (TMMCs) and linalyl acetate (LMMCs).

The development of TMMCs and LMMCs) comprised of mixing 50 mL of 2.5 wt.% ethylene maleic anhydride copolymer (EMA aqueous solution) with 5.0 g of urea, 0.5 g of ammonium chloride, 0.5 g resorcinol, and 200 mL of deionized water using the overhead mechanical stirrer for 5 min under 200 to 320 rpm until homogenization. Then, the pH of the solution was measured using the pH measurement device and maintained in the range of 2.5 to 3.5 using HCl and/or NaOH. After obtaining the desired pH, 50 mL of the self-healing agent (linalyl acetate or tung oil) was added dropwise separately to form two different emulsions under the stirring rate of 900 rpm. The system was then allowed to stabilize for 10 to 20 min. Later, the solution was placed in a water bath and heated to a temperature of 55 °C. At this stage, 13 g of 37 wt.% of aqueous formaldehyde was added to the solution under a stirring rate of 900 rpm. The temperature of the system was maintained at 55 °C for 4 h with continuous stirring of 900 rpm. Finally, the obtained solution was filtered at room temperature under a vacuum and rinsed with water. Finally, it was allowed to dry at room temperature, resulting in the formation of TMMCs and LMMCs. Figure 2 schematically illustrates the contributing steps to develop TMMCs and LMMCs.

### 2.3. Preparation of Substrates and Coatings

The substrates were cleaned by a grinding and polishing machine (Forcipol 1V, Metkon, Bursa, Turkey) using SiC abrasive papers with grades of 80 and 120. The cleaned substrates were washed with distilled water and dried with air. Then, the substrates were cleaned with acetone or ethanol to remove any contaminants on their surface. Five different coatings were prepared for comparison and analysis: (a) pure epoxy coatings-reference coatings (b) epoxy with 3 wt.% tung oil-TMCOATs-3, (c) epoxy with 5 wt.% tung oil-TMCOATs-5, (d) epoxy coatings with 3% linalyl acetate-LMCOATs-3 and (e) epoxy coatings with 5 wt.% linalyl acetate-LMCOATs-5. The TMMCs (3 wt.% and 5 wt.%) and LMMCs (3 wt.% and 5 wt.%) were separately and uniformly dispersed into the epoxy matrix and finally individually coated on the steel substrates applying a doctor blade technique to develop TMMCs reinforced smart coatings (TMCOATs), and LMMCs reinforced smart coatings (LMCOATs), respectively. More precisely, the coatings were prepared by mixing separately epoxy with TMMCs and LMMCs and hardener (the epoxy amount is 5 times the hardener amount). The mixture was then sonicated in the sonication machine for 5 min to ensure good dispersion of the TMMCs/LMMCs with the epoxy and hardener mixture and to ensure the removal of the air bubbles before applying the coating on the substrate. Finally, coatings were applied on the steel substrates using the doctor blade technique and cured at room temperature for 48 h.

### 2.4. Characterization

The morphology and composition of the prepared TMMCs and LMMCs were studied by a field emission scanning electron microscope (FE-SEM-Nova Nano-450), coupled with Energy Dispersive X-ray (EDX) analyzer. Thermal stability of TMMCs and LMMCs and the prepared coatings (reference, TMCOATs, and LMCOATs) was investigated by thermal gravimetric analysis (TGA, 4000, Perkin Elmer, Waltham, MA, USA). The test was conducted in a temperature range of 40–600 °C with an applied heating rate of 20 °C/min. Fourier-transform infrared spectroscopy (FTIR) test was conducted on both types of microcapsules (TMMCs and LMMCs) and the developed coatings (reference, TMCOATs, and LMCOATs) applying FTIR Frontier instrument (Frontier-MIR, Perkin Elmer, Waltham, MA, USA). The main concept of the test is the ability of each bond in the tested substance to absorb infrared radiation at a specific absorption frequency range, which acts as a fingerprint for each bond [13]. The FTIR analysis was carried out with a wavenumber range of 4000 to 500 cm^−1^. Furthermore, a particle size test was undertaken using the particle size analyzer (Master sizer 2000/Malvern, Netherlands) to confirm the average size of the prepared TMMCs and LMMCs and to quantify the occupied volume of each particle size. Electrochemical impedance spectroscopy (EIS) was performed using Gamry device 3000 (Reference 3000, Potentiostat/Galvanstate, Warminster, PA, USA) to examine the corrosion resistance of the developed coatings when subjected to controlled mechanical damage in 0.1 M NaCl solution. During the test, the developed coatings and a graphite rod were used as the working and counter electrode, respectively, while the reference electrode was a narrow tube containing KCl aqueous. The Gamry cell was filled with 0.1 M NaCl. This corrosion test was carried out at room temperature at the frequency range of 0.01 to 100,000 Hz with an AC voltage of 10 mV.

## 3. Results and Discussion

### 3.1. Morphological Analysis of the Synthesized Microcapsules

**Figure 3:**
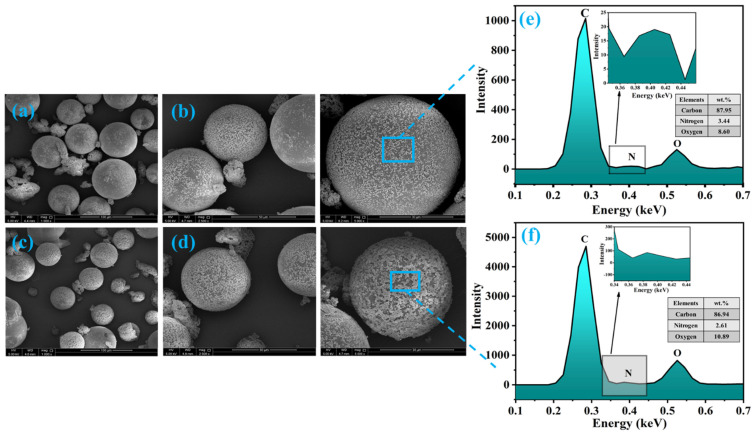
Surface morphology of the microcapsules at different resolution (**a**,**b**) urea-formaldehyde microcapsules loaded with linalyl acetate-LMMCs (**c**,**d**) urea-formaldehyde microcapsules loaded tung oil-TMMCs d (**e**,**f**) EDX analysis of LMMCs and TMMCs.

The morphology of synthesized microcapsules (TMMCs and LMMCs) and loading of self-healing agents i.e., tung oil (TO) and linalyl acetate (LA), into urea-formaldehyde microcapsules (UFMCs) was analyzed by field emission scanning electron microscopy (FE-SEM). Figure 3a,b and Figure 3c,d depict the micrographs of LMMCs and TMMCs containing linalyl acetate and tung oil, respectively. The micrographs show the globular structure of the capsules with almost negligible inter-capsular bonding. It can be noticed from the globular shape that the LA and TO are successfully loaded into urea-formaldehyde microcapsules. A slight rough outer surface is observed in LMMCs compared to TMMCs microcapsules, which enhances the mechanical interlocking of these capsules with the coating matrix. Furthermore, a size variation within both types of microcapsules is observed, which can be due to varying shear force experienced across the center and peripherals of oil drops because of mechanical stirring [20,27]. The micrographs also reveal few punctured microcapsules in the TMMCs, which represents their enhanced sensitivity when compared to LMMCs. The morphological analysis of the synthesized microcapsules is in agreement with already reported literature [17,28,29]. In order to have more insight into the developed microcapsules, the EDX analysis is conducted, and the results are presented in Figure 3e,f. Carbon, nitrogen, and oxygen are the main elements observed in the EDX analysis, representing the elemental composition of urea-formaldehyde microcapsules, tung oil, and linalyl acetate. The EDX analysis confirms the loading of LA and TO into LMMCs and TMMCs, respectively.

### 3.2. Thermal Stability

**Figure 4:**
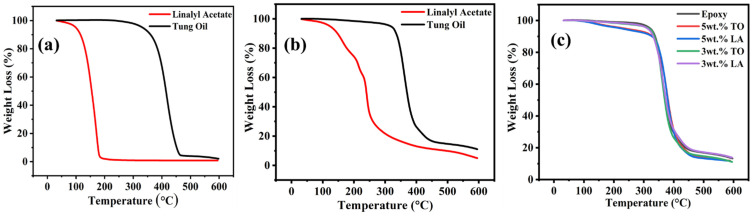
TGA results; (**a**) pure LA and TO, (**b**) urea-formaldehyde microcapsules loaded with linalyl acetate-LMMCs and urea-formaldehyde microcapsules loaded with tung oil-TMMCs, (**c**) Reference coatings and TMCOATs and LMCOATs.

TGA results of LMMCS and TMMCs and the developed coatings (reference coatings, TMCOATs, and LMCOATs) using two different amounts (3 wt.% and 5 wt.%) of TO and LA are presented in Figure 4a–c. For an exact comparison, the TGA results of pure TO and LA are illustrated in Figure 4a. The TGA quantifies the mass degradation of samples while undergoing continuous heating. Generally, the thermal degradation of the material with a biomass pyrolysis process happens in three stages [30], as shown in Figure 4b,c. In the first stage, a weight reduction of the material occurs due to the evaporation of high boiling liquids and moisture in the compound. However, the second and third stages reflect active and passive pyrolysis [30]. The second stage is a critical stage because it is the main weight reduction stage as a result of vapor and gas releasing due to the decomposition of C-O and C-C bonds. Besides, the third stage involves the degradation of the components that contain carbon and remain at the highest temperature levels, which is called char residue [31,32]. Hence, LMMCS, TMMCs, and the developed coatings (reference coatings, TMCOATs, and LMCOATs) show a continuous decrease in the weight as the temperature increases up to 600 °C. Figure 4b shows the TGA results of TMMCs and LMMCs. The initial weight loss (50–100 °C) occurs due to moisture release. Furthermore, complete degradation of LMMCs at a temperature ~250 °C is observed because of the evaporation of linalyl acetate (boiling point of linalyl acetate is ~220 °C). However, TMMCs show a complete weight loss close to 400 °C because of the evaporation of the tung oil (boiling point of tung oil is 375 °C). As a comparison, TMMCs demonstrate better thermal stability compared to the LMMCs. These findings are consistent with previous studies [20,33]. Figure 4c represents the TGA analysis of reference coatings, TMCOATs, and LMCOATs containing different amounts of TMMCs and LMMCs. The results indicate that TMCOATs and LMCOATs show high similarity with the thermal behavior of the reference coatings due to the high percentage of epoxy in them (approximately 95–97% is epoxy). A close analysis of the TGA curves of developed coatings indicates that both the self-healing agents (TO and LA) have been successfully loaded into TMMCs and LMMCs, respectively. The development of the LMCOATs and TMCOATs contributes to achieving thermal stability up to 450 °C, as shown in Figure 4c. Moreover, the increase of the microcapsules in the coatings did not show an observable enhancement in the thermal stability of the coatings.

### 3.3. Particle Size Analysis

**Figure 5:**
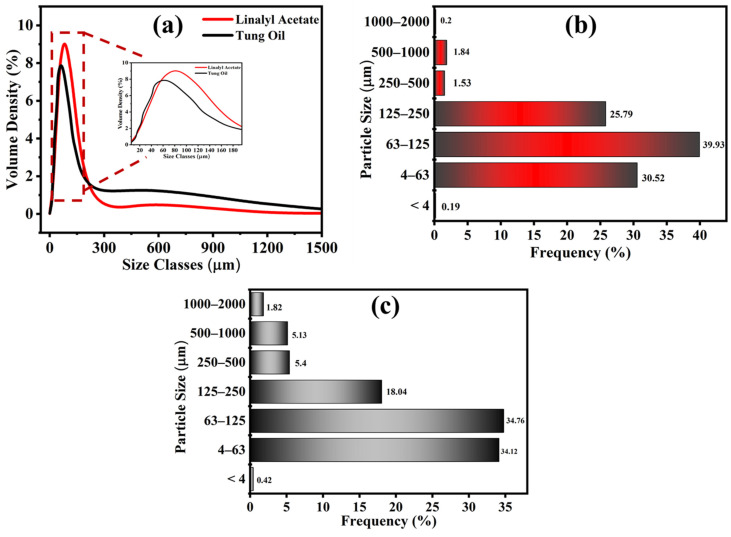
Particle size analysis. (**a**) Gaussian particle size distribution in LMMCs and TMMCs, (**b**) bar chart showing distribution of LMMCs and (**c**) bar chart showing distribution of TMMCs.

Particle size analysis was carried out to confirm the particle size distribution of synthesized TMMCs and LMMCs, as shown in Figure 5. As can be seen that the particle size of the TMMCs and LMMCs is in the range of 0.03 to 600 µm, applying a stirring rate of 900 rpm (Figure 5a). It is worth mentioning that, as the stirring rate increases, the microcapsules particle size decreases [34,35,36]. The higher stirrer speed causes more splitting of the drops of the self-healing agent (tung oil and linalyl acetate), which results in finer microcapsules. Figure 5a illustrates that the majority of LMMCs and TMMCs microcapsules have the same particle size (79.5 µm). A particle size distribution bar chart is plotted to present the percentage of each particle size range that exists in the studied samples. Figure 5b,c shows the particle size distribution of LMMCs and TMMCs. As can be seen in Figure 5b, the least dominant LMMCs sizes are the particles with sizes less than 4 µm and the ones with size range of 1000 to 2000 µm with the contribution of 0.19% and 0.2% respectively. However, the most dominant microcapsule size is located in the range of 63 to 125 um with an occupation percentage of 39.93. On the other hand, Figure 5c shows that the TMMCs with dimensions less than 4 µm occupy only 0.42%, which are the least dominant microcapsules. Moreover, the TMMCs with sizes of 63 to 125 µm are the most dominant ones. This representation confirms the direct effect of the stirring speed on the particle size distribution. The reason for having different microcapsule sizes is the difficulty of controlling the distribution of the solution in the container during the stirring. The solution, which is located closer to the stirrer, experiences more agitation, resulting in microcapsules with smaller particle sizes. In contrast, the solution located away from the stirrer undergoes relatively weak agitation leading to unfavorable splitting and thus a larger particle size. This can be further noticed that synthesis of both LMMCs and TMMCs under identical experimental conditions (constant stirring speed 900 rpm and temperature 55 °C) has resulted in the same particle size range (63–125 um) with a similar contribution percentage (Figure 5b,c).

### 3.4. FTIR Analysis

**Figure 6:**
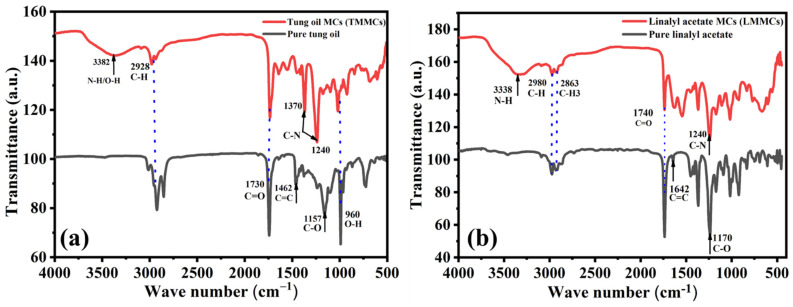
FTIR spectra of (**a**) pure linalyl acetate and LMMCs (**b**) pure tung oil and TMMCs.

FTIR analysis was conducted to confirm the loading of linalyl acetate and tung oil into urea-formaldehyde microcapsules. Figure 6a,b shows the FTIR spectra of TMMCs and LMMCs. In Figure 6a, the wideband in the TMMCs at 3382 cm^−1^ can be ascribed to the N-H bond in urea-formaldehyde and the O-H bond due to the presence of moisture. Moreover, the 1370 and 1240 cm^−1^ sharp peaks in the TMMCs indicate the presence of C-N bond, which is one of the unique bonds in the urea-formaldehyde structure. Furthermore, the peaks at 1462 and 1157 cm^−1^ reflect the presence of C=C and C-O in tung oil, respectively, which are distinctive bonds that represent the tung oil structure. However, the peaks at 2928, 1730, and 980 cm^−1^ in pure tung oil and TMMCs are due to the presence of C-H, C=O, and O-H bonds, respectively. These shared sharp peaks in the FTIR spectra indicate the efficient loading of tung oil in urea-formaldehyde microcapsules. These findings are consistent with previous studies [20,27,37].

In FTIR spectra of LMMCs, as shown in Figure 6b, the peaks at 2980, 2863, and 1740 cm^−1^ reflect the presence of C-H, C-H_3_, and C=O bonds, respectively. These peaks coexist between the pure linalyl acetate and LMMCs, which indicates the successful loading of linalyl acetate in urea-formaldehyde microcapsules. At the same time, the broad peak at 3338 cm^−1^ represents the N-H bond in urea-formaldehyde, confirming the presence of the urea-formaldehyde microcapsules structure. Moreover, the peak at 1642 cm^−1^ and the dominant peak at 1170 cm^−1^ in the pure linalyl acetate show the presence of C=C and C-O bonds, respectively. However, the sharp peak at 1240 cm^−1^ in LMMCs shows the presence of the C-N bond, indicating the contribution of urea-formaldehyde in the structure of LMMCs. These results are consistent with the results of previous related studies [20,37].

### 3.5. Self-Healing of Smart Coatings

**Figure 7:**
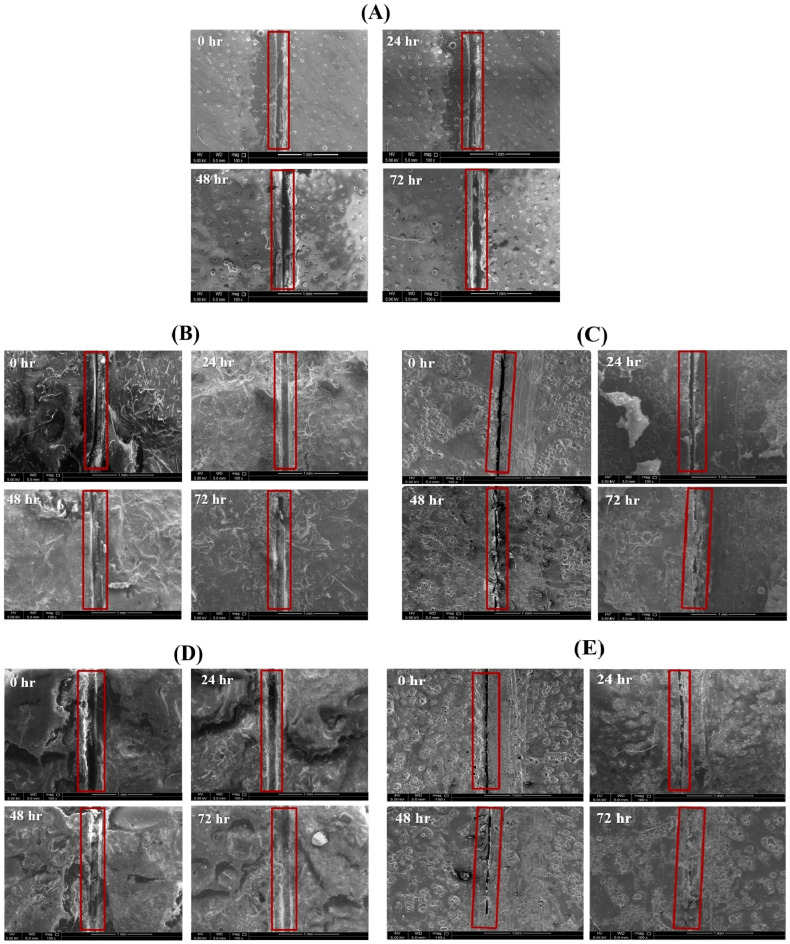
Self-healing analysis of smart coatings at various time intervals, (**A**) Reference coating (plain epoxy), (**B**) 3 wt.% TMCOATs, (**C**) 3 wt.% LMCOATs, (**D**) 5 wt.% LMCOATs and (**E**) 5 wt.% TMCOATs. The used scale is 1 mm.

**Figure 8:**
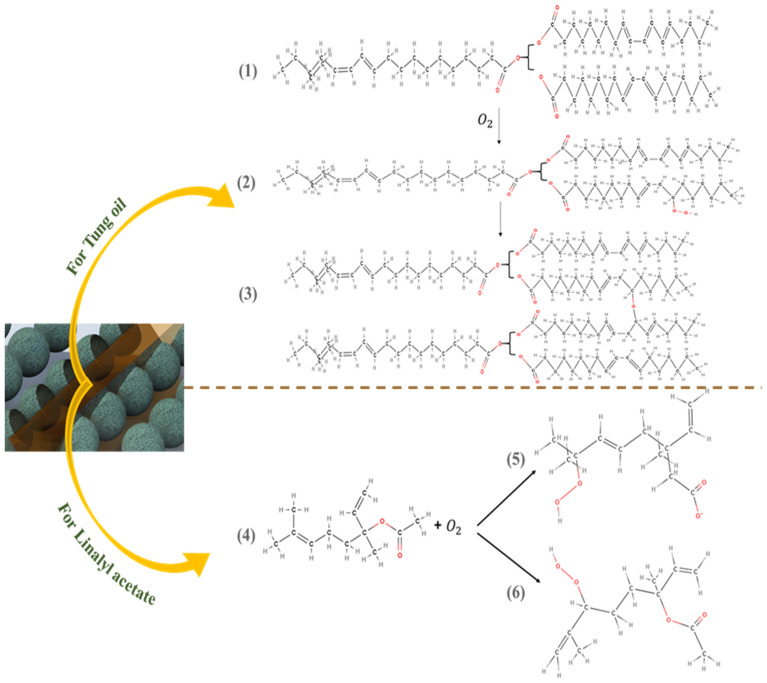
Self-healing detailed reactions of linalyl acetate and tung oil.

Figure 7A–E demonstrates the self-healing ability of reference coating, TMCOATs and LMCOATs containing different concentrations of TMMCs (3 wt.% and 5 wt.%) and LMMCs (3 wt.% and 5 wt.%). During the controlled damage (scratched area highlighted in red), the TMMCs and LMMCs are ruptured and release their loaded self-resented as fatty acids [38]. After the release of the tung oil, the polymerization process takes place in fatty acids by free radical or cationic mechanism [39]. The polymerization process results in an elastic film that is stable and difficult to flow or deform and has a heavier texture as it absorbs air [40]. In tung oil, the polymerization takes place by a free radical mechanism. The detailed oxygenation reactions of linalyl acetate and tung oil due to their exposure to atmospheric air after scratch are shown in Figure 8. Drying oils (such as tung oil) polymerize without the aid of any catalyst. In this situation, an oxidative polymerization of tung oil (1) takes place with the exposure into the air, which heals the matrix’s crack. They contain triglycerides, which have high levels of polyunsaturated fatty acids that contain one or two methylene groups (bis-allylic). These allylic positions are very sensitive to oxidation by molecular oxygen in air, which is known as “autoxidation”. The autoxidation process leads to the formation of hydroperoxides (2) and the formation of bonds between different triglyceride molecules. A hard film is formed when a cross-link between triglyceride (3) molecules takes place, which forms an oxypolymerization network. Similarly, the linalyl acetate is also oxidized in the air after its release from LMMCs because of the oxidizable positions in its structure. In a similar manner, during the exposure of linalyl acetate into atmospheric air, it produces different compounds such as hyperoxides, epoxide, and alcohol [20,41,42], resulting in the formation of a protective or passive layer isolating the damaged area from corrosive medium [8]. As shown in Figure 8, when the oxygen reacts with linalyl acetate (4), a mixture of 7-hydroperoxy-3,7-dimethylocta-1,5-diene-3-yl-acetate (5) and 6-hydroperoxy-3, 7-dimethylocta-1,7-diene-3-yl acetate (6) provides a thin passive film that induces passivation of the metal [43,44].

As can be seen in Figure 7, the coatings that contain microcapsules show an effective self-healing ability with time, compared with the reference coating that did not show any healing due to the absence of the self-healing agent. As a comparison, both TMCOATs and LMCOATS coatings containing 5 wt.% microcapsules show a more efficient self-healing ability than 3 wt.% due to the increased number of microcapsules, which allows the release of a higher amount of self-healing agents to fill the defected area.

### 3.6. Electrochemical Impedance Spectroscopy (EIS)

**Figure 9:**
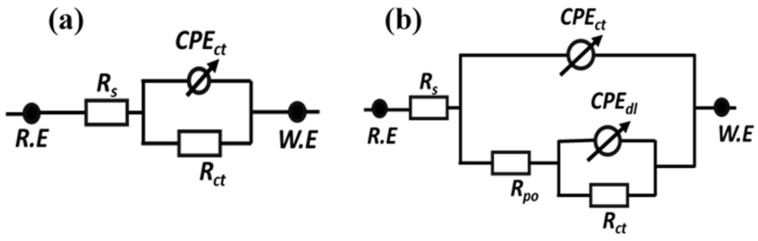
Equivalent circuits used to fit the electrochemical impedance spectroscopy data (**a**) reference coatings (**b**) TMCOATs and LMCOATs.

The EIS technique has been utilized as an effective method for studying the corrosion performance of the reference coatings, TMCOATs and LMCOATs, when immersed in 0.1 M NaCl solution for four days after an artificial scratch. Figure 9a,b presents the equivalent circuits used for fitting the experimental results and quantification of electrochemical parameters. A one-time constant equivalent electrical circuit, which is commonly used for analyzing reference coatings, is depicted in Figure 9a. In contrast, a two-time constant equivalent circuit as shown in Figure 9b was used for analyzing the smart coatings (TMCOATs and LMCOATs). The elements of the equivalent circuits can be described as the solution resistance of 0.1 M NaCl solution (*R_s_*) and pore resistance of coatings (*R_po_*); constant phase elements (*CPE_ct_* and *CPE_dl_*) are used instead of a pure capacitor for the accurate acknowledge of system capacitance due to charge transfer. In addition, double-layer capacitance and charge transfer resistance of the coating (*R_ct_*) were calculated from impedance plots.

**Figure 10:**
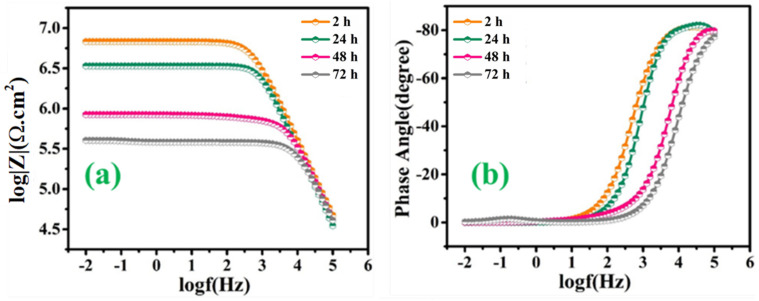
EIS analysis of reference coatings (**a**) bode graph (**b**) phase angle graph.

**Figure 11:**
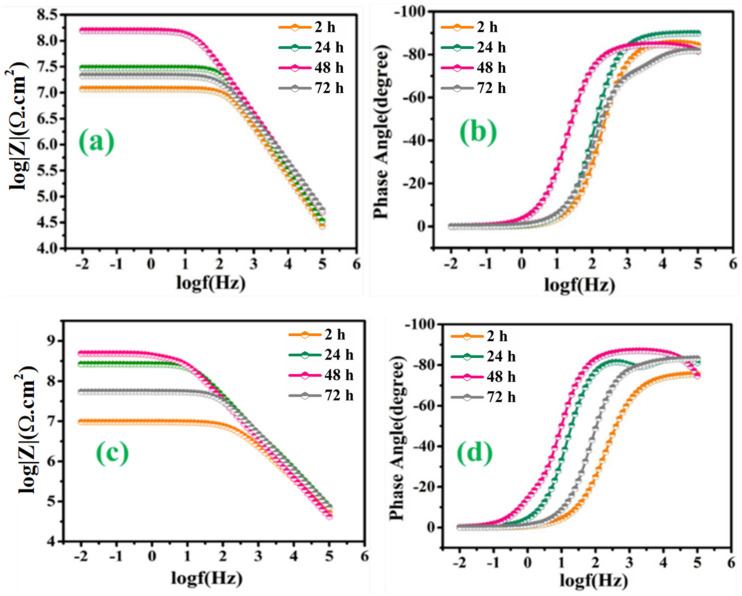
TMCOATs; (**a**) Bode graph of 3 wt.% TMMCs (**b**) phase angle graph of 3 wt.% TMMCs (**c**) Bode graph of 5 wt.% TMMCs (**d**) phase angle graph 5 wt.% TMMCs.

Figure 10a,b represents the bode and phase angle plots of reference coatings. As can be seen, in the high-frequency domain, the impedance results present a capacitive behavior. However, in the low-frequency domain, the *R_ct_* of reference coating was measured to be 6.51 × 10^6^ Ω∙cm^2^ after 2 h of immersion, which started decreasing gradually for the successive duration of immersion for 72 h (1.99 × 10^5^ Ω∙cm^2^). The reduction in the *R_ct_* value of reference coatings confirms high corrosion activity at the substrate coating interface due to the absence of a self-healing agent, which can delay the corrosion activity. Similarly, Figure 11a–d demonstrates the magnitude and phase angle plots of TMCOATs after various immersion times (2, 24, 48, 72 h). An increase in the capacitive response in the mid-high frequency domain compared to the reference coating is due to the presence of a self-healing agent (tung oil), which delays the corrosion activity at the scratched zones. Moreover, the bode graph shows an increase in the early stages of *R_ct_* values of TMCOATs with respect to immersion time at low-frequency range that confirms an improved resistance to corrosion at the coating interface. The resistance of TMCOATs with 3 wt.% TMMCs increased by an order (*R_ct_* = 1.54 × 10^8^ Ω∙cm^2^) after the third day of immersion and displayed a slight decrease after the fourth day depicted in Figure 11a. The decrease after the fourth day can be associated with the penetration of electrolyte solution through the newly developed passive layer at the scratched zone of the coatings. Furthermore, the bode graph of the TMCOATs with 5 wt.% TMMCs showed similar corrosion-resistant behavior with respect to immersion time. Comparatively more stable and higher *R_ct_* values were observed after the second and third day of immersion, which reflected the high concentration of the TMMCs in the coating matrix.

**Figure 12:**
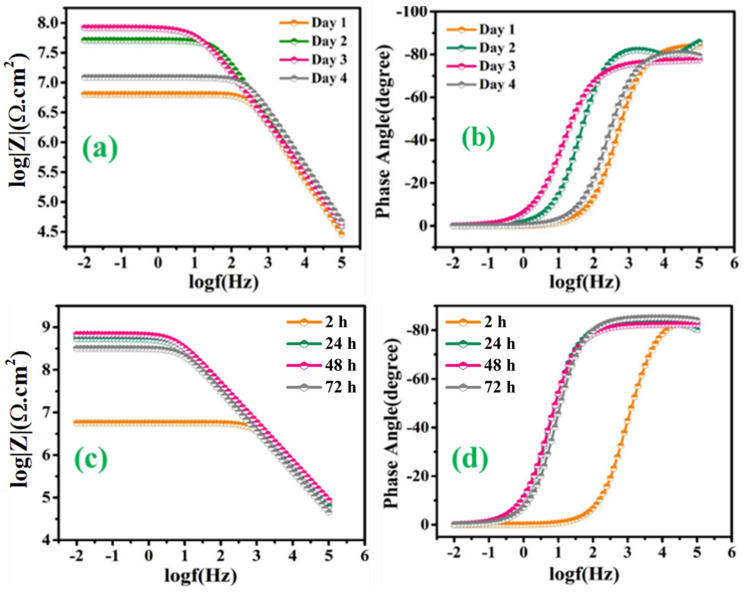
LMCOATs; (**a**) Bode graph of 3 wt.% LMMCs, (**b**) phase angle graph of 3 wt.% LMMCs, (**c**) Bode graph of 5 wt.% LMMCs, (**d**) phase angle graph 5 wt.% LMMCs.

On the other hand, Figure 12a–d depicts the EIS spectra, after the immersion of the scratched LMCOATs for different exposure times: 2, 24, 48, and 72 h. The LMCOATs showed a similar increasing trend of the coating resistance at the early stages of the scratch, as observed in the TMCOATs. Furthermore, high stability was observed in the *R_ct_* values of LMCOATs. The *R_ct_* of LMCOATs containing 5 wt.% LMMCs after 48 h (7.54 × 10^8^ Ω∙cm^2^) showed a three-order increment compared to the corresponding value of reference coatings. The overlapping of the bode spectra after the immersion of 24, 48, 72 h in the low-frequency region reflects the superior barrier properties and the uniformity in the passive layer formation at the scratched area of 5 wt.% LMCOATs. As bubble-free polymeric coatings are nearly impossible to develop, bubbles act as a double-edged weapon because it promotes the oxypolymerization of tung oil and linalyl acetate in the scratched area, which fills the exposed area of the scratched coatings and prevents the interaction of the substrate with the harsh corrosive media [37].

Furthermore, the TMCOATs and LMCOATs with 3 wt.% of microcapsules showed lower resistance compared to 5 wt.%, which can be because of the insufficient amount of self-healing species needed to heal the scratched part of the coatings. Moreover, the smaller particle size of the as-synthesized microcapsules (as mentioned in detail in Section 3.3) helped to obtain an excessive distribution across the coating matrix, which in return resulted in the higher probability of releasing of self-healing agents upon mechanical damage because of a numerous of microcapsules to fill the damaged area [27].

In conclusion, more stable and superior coating resistance was observed in the LMCOATs with 5 wt.% of LMMCs, which reflects the efficient polymerization of linalyl acetate at the scratched area. Moreover, it was noticed from the high *R_ct_* values after 72 h that 5 wt.% of the microcapsules in the coating matrix results in continuous and almost defect-free passive layer formation compared to that of 3 wt.% microcapsules.

**Figure 13:**
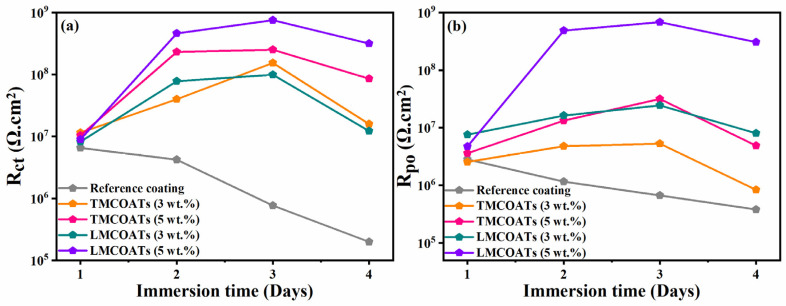
Variation of electrochemical parameters with immersion time; (**a**) charge transfer resistance and (**b**) pore resistance of the reference, TMCOATs and LMCOATs.

Figure 13 depicts the quantified values of *R_ct_* and *R_po_* of the TMCOATs and LMCOATs throughout immersion, respectively. The *R_ct_* values of the reference coatings show a continuous decrease over the immersion period, which reflects the initiation of the corrosion activity at the exposed steel substrate. On the contrary, the TMCOATs and LMCOATs showed incremental *R_ct_* values at 2, 24 and 48 h, and then showed a slight decrease. This increase in the magnitude of *R_ct_* in TMCOATs and LMCOATs confirms the negligible corrosion activity at the substrate coating interface. Moreover, the improvement in *R_ct_* is also consistent with the increase in *R_po_* of TMCOATs and LMCOATs, which shows the efficient healing capability of TMMCs and LMMCs.

**Figure 14:**
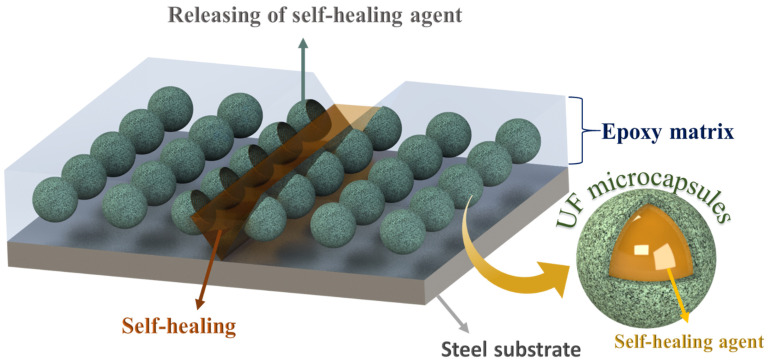
Self-healing mechanism of smart coatings TMCOATs and LMCOATs.

The epoxy matrix reinforced with microcapsules containing self-healing agents when exposed to external physical pressure or crack causes some of the microcapsules along the scratch to rupture and release the self-healing species in the damaged area. The self-healing species, when exposed to the environment, polymerizes and restricts the contact of the bare steel substrate with the corrosive media. The proposed system has an autonomous self-healing mechanism, which repairs the damage on its own without requiring any external physical interference or aid. In the current study, the epoxy matrix reinforced with microcapsules containing TO and LA acts as self-healing carriers. Figure 14 illustrates the self-healing mechanism of the proposed system. The artificial mechanical damage in the coat causes rupture of the TMMCs and LMMCs, leading to the release of TO and LA in the damaged area. Moreover, TO and LA tends to polymerize in the presence of surrounding oxygen causing oxypolymerization, which results in the formation of a thin passive layer in the scratch zone. Hence, the formed film delays or limits the electrolyte from reaching the substrate and thus results in improved corrosion resistance. These findings are also consistent with previous studies [20,27,45,46].

## 4. Conclusions

Self-healing smart coatings (TMCOATs and LMCOATs) containing different concentrations of urea-formaldehyde microcapsules (3 wt.% and 5 wt.%) loaded with tung oil (TMMCs) and linalyl acetate (LMMCs) were respectively developed and characterized. The developed smart coatings demonstrate superior corrosion resistance when compared to the reference coatings. The self-healing characteristics of smart coating are sensitive to the concentration of TMMCs and LMMCs. Comparatively, LMCOATs exhibit more stable barrier properties reaching ~1Gohm, when compared with other smart coatings depicting their usefulness for some industrial applications.

## Data Availability

The data presented in this study are available on request from the corresponding author.
